# Mechanical Characterization of Hybrid Vesicles Based on Linear Poly(Dimethylsiloxane-b-Ethylene Oxide) and Poly(Butadiene-b-Ethylene Oxide) Block Copolymers

**DOI:** 10.3390/s16030390

**Published:** 2016-03-18

**Authors:** Jeffery Gaspard, Liam M. Casey, Matt Rozin, Dany J. Munoz-Pinto, James A. Silas, Mariah S. Hahn

**Affiliations:** 1 Department of Chemical Engineering, Texas A&M University 3122 TAMU, College Station, 77840 TX, USA; jeffery.gaspard@hotmail.com (J.G.); james.a.silas@gmail.com (J.A.S.); 2 Department of Chemical Engineering, University of Massachusetts, Amherst, 01003 MA, USA; lmcasey@umich.edu (L.M.C.); mrozin@ucsd.edu (M.R.); 3 Engineering Science Department, Trinity University, San Antonio, 78212 TX, USA; dmunozpi@trinity.edu; 4 Department of Biomedical Engineering, Rensselaer Polytechnic Institute, 110 8th Street, Biotech 2434, Troy, 12180 NY, USA

**Keywords:** polymersomes, poly(butadiene), poly(dimethylsiloxane), hybrid vesicles

## Abstract

Poly(dimethylsiloxane-ethylene oxide) (PDMS-PEO) and poly(butadiene-b-ethylene oxide) (PBd-PEO) are two block copolymers which separately form vesicles with disparate membrane permeabilities and fluidities. Thus, hybrid vesicles formed from both PDMS-PEO and PBd-PEO may ultimately allow for systematic, application-specific tuning of vesicle membrane fluidity and permeability. However, given the relatively low strength previously noted for comb-type PDMS-PEO vesicles, the mechanical robustness of the resulting hybrid vesicles must first be confirmed. Toward this end, we have characterized the mechanical behavior of vesicles formed from mixtures of linear PDMS-PEO and linear PBd-PEO using micropipette aspiration. Tension *versus* strain plots of pure PDMS_12_-PEO_46_ vesicles revealed a non-linear response in the high tension regime, in contrast to the approximately linear response of pure PBd_33_-PEO_20_ vesicles. Remarkably, the area expansion modulus, critical tension, and cohesive energy density of PDMS_12_-PEO_46_ vesicles were each significantly greater than for PBd_33_-PEO_20_ vesicles, although critical strain was not significantly different between these vesicle types. PDMS_12_-PEO_46_/PBd_33_-PEO_20_ hybrid vesicles generally displayed graded responses in between that of the pure component vesicles. Thus, the PDMS_12_-PEO_46_/PBd_33_-PEO_20_ hybrid vesicles retained or exceeded the strength and toughness characteristic of pure PBd-PEO vesicles, indicating that future assessment of the membrane permeability and fluidity of these hybrid vesicles may be warranted.

## 1. Introduction

Block copolymer-based vesicles have been instrumental in developing mechanically robust nano/micron-scale reactors, drug carriers, and sensors [1,2,3,4,5,6,7,8,9]. For each of these applications, membrane fluidity (e.g., so as to achieve desired localization of molecular recognition elements) and membrane permeability (e.g., so as to achieve desired reactant/product concentrations within the vesicle) are as critical to device performance as membrane expansion modulus, strength, and toughness. In the present manuscript, we propose to fabricate vesicles formed from graded mixtures of two distinct diblock copolymers toward the long-term goal of developing polymersomes with tailorable membrane permeabilities and fluidities.

**Figure 1:**
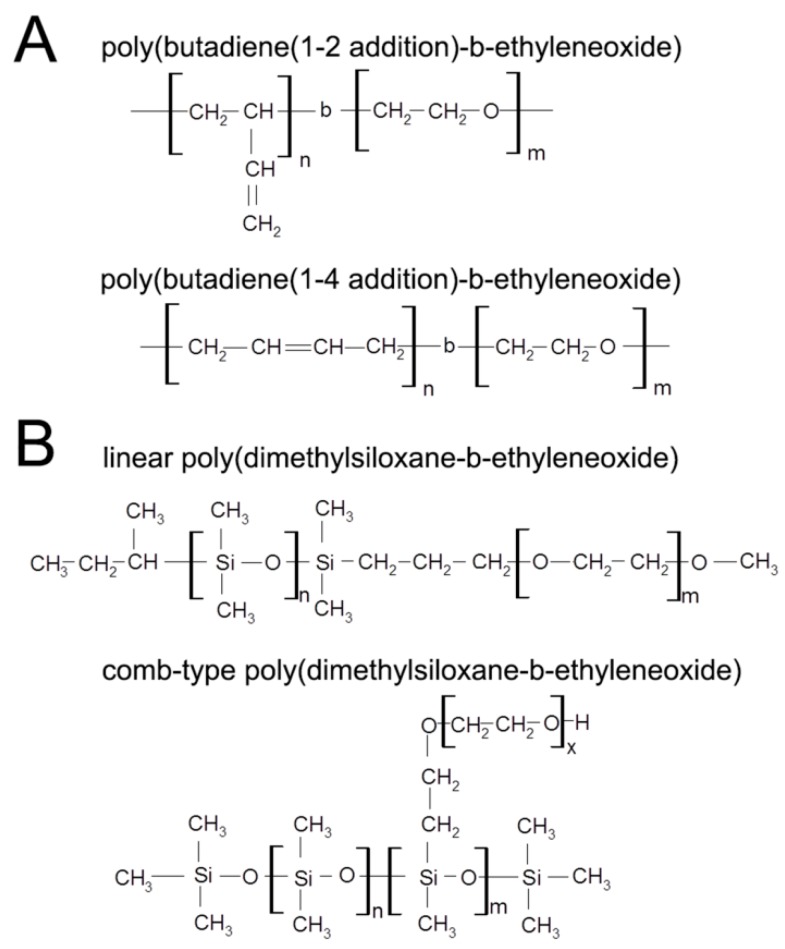
Structures (**A**) of poly(butadiene(1–2 addition)-b-ethylene oxide) *versus* poly(butadiene(1–4 addition)-b-ethylene oxide); and (**B**) of linear poly(dimethylsiloxane-b-ethylene oxide) *versus* comb-type poly(dimethylsiloxane-b-ethylene oxide). Poly(butadiene(1–2 addition)-b-ethylene oxide) and linear poly(dimethylsiloxane-b-ethylene oxide) were utilized herein for reasons detailed in the main text.

Toward this end, we chose to generate hybrid vesicles from linear poly(butadiene(1-2 addition)-b-ethylene oxide) (PBd-PEO) (Figure 1A) [9,10,11,12,13,14,15,16,17] and linear poly(dimethylsiloxane-b-ethylene oxide) (PDMS-PEO) (Figure 1B) [18,19,20,21], two diblock copolymers that separately form vesicles of markedly different membrane permeabilities and fluidities. Specifically, a standardly employed PBd-PEO copolymer (PBd_46_-PEO_30_) results in vesicle membranes with a water permeability of ≈3.1 µm/s [11] and an estimated lateral diffusion coefficient of ≈0.01 µm^2^/s [22]. In contrast, no signs of physical separation between intravesicle and extravesicle water fractions were observed by Pulsed Field Gradient (PFG)-NMR for vesicles formed from linear PEO_15_-PDMS_15_-PEO_15_ triblock copolymers [21]. These PFG-NMR results indicate that PEO_15_-PDMS_15_-PEO_15_ vesicle membranes present a minimal barrier to the diffusion of water. In addition, lateral diffusion coefficients for vesicles formed from linear PDMS_22-31_-PEO_6-9_ diblock copolymers range from 4.6–6.0 µm^2^/s [20], over 100-fold greater than the lateral diffusivities estimated for PBd_46_-PEO_30_ membranes [22]. As such, vesicles fabricated from mixtures of PBd-PEO and PDMS-PEO may display graded membrane fluidities and permeabilities between those of the pure component vesicles.

That said, the mechanical properties of PDMS-PEO/PBd-PEO vesicles must first be assessed to ensure that these hybrid vesicles retain necessary membrane expansion modulus, strength, and toughness. This is particularly important given that the mechanical properties of vesicles formed from linear PDMS-PEO have not previously been evaluated. In addition, vesicles formed from comb-type PDMS-PEO (Figure 1B) appear to display a critical tension of ≈7.6 mN/m and an average critical strain under 8% [7,11,23,24,25], values well below the ≈16 mN/m critical tension and ≈20% critical strain associated with vesicles formed from linear PBd-PEO of similar molecular weight [13,24]. The present manuscript therefore investigates the mechanical behavior of hybrid vesicles formed from graded mixtures of linear PDMS-PEO and linear PBd-PEO diblock copolymers under conditions of high tension.

## 2. Materials and Methods

Linear poly(butadiene(1-2 addition)-b-ethylene oxide) (PBd_33_-PEO_20_; total M_n_ ≈ 2700 g/mol, PBd M_n_ ≈ 1800 g/mol, PEO M_n_ ≈ 900 g/mol; PI = 1.04; 95% 1–2 addition) and linear poly(dimethylsiloxane-b-ethylene oxide) (PDMS_12_-PEO_46_; total M_n_ ≈ 3100 g/mol, PDMS M_n_ ≈ 1000 g/mol, PEO M_n_ ≈ 1800 g/mol; PI = 1.12) block copolymers were purchased from Polymer Source Inc. Sucrose (ACS reagent grade), phosphate buffered saline (PBS), sodium chloride (ACS reagent grade), and bovine serum albumin (BSA) were purchased from Fisher Scientific. Dichloromethane (DCM; anhydrous, 99.9%) was purchased from Acros Organics. Nile Red (97%) was purchased from Sigma-Aldrich.

### 2.1. Formation of Polymersomes

Seven distinct block copolymer vesicles were investigated: (1) 100 wt% PDMS_12_-PEO_46_ (100% PDMS); (2) 95 wt% PDMS_12_-PEO_46_: 5 wt% PBd_33_-PEO_20_ (95% PDMS); (3) 75 wt% PDMS_12_-PEO_46_: 25 wt% PBd_33_-PEO_20_ (75% PDMS); (4) 50 wt% PDMS_12_-PEO_46_: 50 wt% PBd_33_-PEO_20_ (50% PDMS); (5) 25 wt% PDMS_12_-PEO_46_: 75 wt% PBd_33_-PEO_20_ (25% PDMS); (6) 10 wt% PDMS_12_-PEO_46_: 90 wt% PBd_33_-PEO_20_ (10% PDMS); and (7) 100 wt% PBd_33_-PEO_20_ (0% PDMS). To fabricate each block copolymer vesicle type, thin films were first prepared per standard methodologies [12,13,26]. In brief, a 5 mg/mL solution of each block copolymer mixture was prepared in DCM, a solvent which is able to effectively solubilize both PDMS-PEO and PBd-PEO. Following transfer of 50 microliters of each solution to separate 5 mL glass vials (surface area of vial bottom ≈0.8 cm^2^), the DCM solvent was allowed to evaporate at room temperature over a period of 8 h. Polymersomes were subsequently formed by rehydration of the block copolymer films at 0.125–0.25 wt % in 1–2 mL of a 300 mOsm/kg sucrose solution for 24 h at 60 °C. In a subset of samples, a small amount of the hydrophobic fluorophore Nile Red dissolved in DCM was added to the film rehydration solution to allow for visualization of the vesicle membrane [1,10].

### 2.2. Optical Microscopy Imaging

Optical microscopy images of vesicle solutions were obtained using a closed-sample chamber system. In order to provide contrast for imaging, vesicles were immersed in a saline solution of ≈320 mOsm/kg, and images were obtained using a Zeiss Axiovert 200M inverted optical microscope coupled to a Zeiss AxioCam MRm camera. Confocal images of Nile Red-containing polymersomes immersed in an external saline solution of ≈310 mOsm/kg were obtained using a Leica TCS SP5 confocal microscope. Samples were imaged in a closed-sample chamber system at 543/600–700 nm emission/excitation using a 63X oil objective lens with a numerical aperture of 1.25. Pictures were taken at a resolution of either 512 × 512 or 1024 × 1024, a refresh rate of 400 Hz, a pinhole size of 100 μm, and a voltage of 700 V for the photomultiplier tube.

### 2.3. Micropipette Aspiration Measurements

To assess the mechanical properties of the synthesized vesicles, room temperature tension-strain curves were generated by vesicle micropipette aspiration under iso-osmotic conditions. Glass micropipettes were prepared using standard techniques [27,28,29] and placed into a custom manometer system. Each pipette tip was coated with BSA to prevent undesired vesicle adhesion. Vesicles were chosen in the 15–50 µm diameter range to avoid error in calculating tension and areal strain. Pressure transducers (Validyne DP45-32) provided measurement of the imposed pressure on a vesicle system, and micromanipulators (Narishige MHW-3) allowed the vesicles to be aspirated and handled.

To generate tension-strain curves, applied pipette pressure was converted into membrane tension (τ) using the following equation [30]: $\tau =\frac{(\Delta P)D_{p}}{4\left(1-\frac{D_{p}}{D_{v}}\right)}$, where Δ*P* is the applied pressure, *D_p_* is the pipette inner diameter, and *D_v_* is the diameter of the exterior vesicle segment. Membrane tension was then plotted against areal strain (α) [30]: $\alpha =\frac{\Delta A}{A}=\left({\left(\frac{D_{p}}{D_{v}}\right)}^{2}-{\left(\frac{D_{p}}{D_{v}}\right)}^{3}\right)\frac{\Delta L}{D_{p}}$, where A is the vesicle surface area and ΔL is the vesicle projection length within the micropipette. From the resulting tension-strain curves, values were estimated for the area expansion modulus as well as other mechanical properties, such as critical tension, critical areal strain, and the cohesive energy density.

### 2.4. Statistical Analyses

All data are reported as mean ± standard error of the mean. Differences in averages among formulations were statistically evaluated using ANOVA followed by Tukey *post hoc* tests (SPSS version 22.0, IBM), with a *p*-value < 0.05 considered significant.

## 3. Results and Discussion

The present manuscript investigates the high tension mechanical behavior of vesicles formed from graded mixtures of linear PDMS_12_-PEO_42_ and PBd_33_-PEO_20_ as a first step toward the long-term goal of enabling broad, application-specific tailoring of polymersome membrane fluidity and permeability. In selecting specific formulations of PBd-PEO and PDMS-PEO to be examined, we considered the following details from the literature. First, the PBd-PEO copolymers utilized in prior vesicle studies have generally been linear in form [9,10,11,12,13,14,15,16,17], and the PBd chains have primarily been 1–2 (as opposed to 1–4) in microstructure (Figure 1A). In contrast, previous studies on PDMS-PEO copolymer vesicles have included both linear and comb-type PDMS-PEO [7,11,18,19,20,23,24,25,31] (Figure 1B). Given the standard use of linear PBd-PEO [9,10,11,12,13,14,15,16,17], we opted to also utilize a linear form of the PDMS-PEO copolymer for the current studies. This choice allowed more facile matching of the hydrophobic and hydrophilic block lengths between the PBd-PEO and PDMS-PEO copolymers, improving the potential for stable vesicle formation from PBd-PEO/PDMS-PEO mixtures.

The length of the hydrophobic segment of the linear PDMS-PEO copolymer was selected based on literature indicating that PDMS-PEO copolymers with short PDMS chain lengths result in vesicles membranes with high lateral diffusivity and high water permeability [20,32]. This is in contrast to the relatively low lateral diffusivity and low water permeability generally presented by PBd-PEO copolymer membranes [11,22]. Specifically, PEO_15_-PDMS_15_-PEO_15_ vesicle membranes have been observed to present a minimal barrier to the diffusion of water [32], and PDMS_22-31_-PEO vesicle membranes display lateral diffusivities ranging from 4.6–6.0 µm^2^/s [20]. Based on these data, the linear PDMS-PEO copolymer formulation PDMS_12_-PEO_46_ was chosen for examination herein. 

The linear, 1–2 addition PBd-PEO formulation was then selected to minimize the difference in hydrophobic and hydrophilic block lengths between the selected PDMS-PEO formulation and the PBd-PEO copolymer. In particular, a scaling relationship between membrane thickness and the molecular weight of the hydrophobic block (M_h_) developed for vesicles formed from linear diblock PDMS-b-poly(2-methyloxazoline) indicates that a PDMS block length of 12 would result in membranes with a hydrophobic layer thickness of ≈7.4 nm [20]. Similarly, PBd_33_-PEO_20_ copolymers are estimated—based on experimentally validated scaling relationships for PBd-PEO vesicles—to produce vesicles with a hydrophobic layer thickness of ≈8 nm [13]. This ≈8 nm thickness is similar to the 7.4 nm estimated for the selected linear PDMS_12_-PEO_46_ vesicles. In terms of the hydrophilic segments, the root mean squared lengths for the hydrophilic blocks of PBd_33_-PEO_20_ and PDMS_12_-PEO_46_ are estimated to be 2.7 nm and 4.1 nm, respectively [33]. Perhaps more importantly, the volume fraction of the hydrophilic component ($f_{hydrophilic}$) of the PBd_33_-PEO_20_ copolymer is 0.33, within the range of 0.25 < $f_{hydrophilic}$ < 0.40 demonstrated for these and many other amphiphilic copolymers to be suitable for vesicle formation [8]. In addition, the PDMS_12_PEO_46_ copolymer displays a $f_{hydrophilic}$ of ≈0.67, consistent with observations that linear PDMS-PEO copolymers with $f_{hydrophilic}$ > 50% can support unilamellar vesicle formation [18,19].

Following selection of copolymer formulations, vesicles were prepared from PDMS_12_-PEO_46_, PBd_33_-PEO_20_, and their mixtures—termed 0% PDMS, 10% PDMS, 25% PDMS, 50% PDMS, 75% PDMS, 95% PDMS or 100% PDMS based on the wt % of PDMS-PEO in the mixture—and analyzed by microscopy and micropipette aspiration.

### 3.1. Vesicle Size and Shape

**Figure 2:**
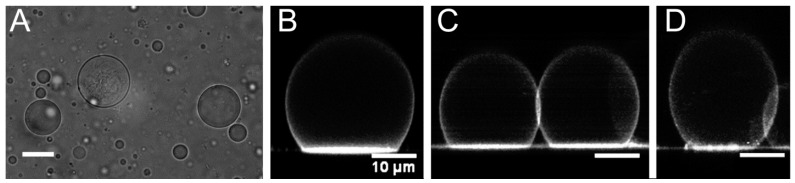
(**A**) A phase contrast image of pure PBd_33_-PEO_20_ vesicles; scale bar = 50 µm. This image is representative of vesicle shape and size for all formulations; Representative confocal images of (**B**) 0% PDMS (pure PBd_33_-PEO_20_); (**C**) 100% PDMS (pure PDMS_12_-PEO_46_); and (**D**) 25% PDMS vesicles prepared in the presence of Nile Red; scale bars in (**B**–**D**) represent 10 µm.

Each vesicle formulation displayed a relatively uniform spherical structure by optical microscopy imaging, and resulting vesicles ranged in size from submicron to greater than 50 µm in diameter (Figure 2A). Phase-contrast imaging indicated that the primary vesicle type formed was unilamellar, although multilamellar vesicles were also observed (Figure 2A, Supplementary Figure S1). Vesicle structure was further visualized by adding the hydrophobic, fluorescent dye Nile Red to the aqueous solution utilized in copolymer film rehydration. Since Nile Red is not significantly water-soluble, it preferentially partitions to hydrophobic regions of vesicle membranes [1,10]. As shown in the confocal images in Figure 2B,C, pure PDMS_12_-PEO_46_ and pure PBd_33_-PEO_20_ appeared to produce relatively spherical, unilamellar vesicles. In addition, both vesicle types formed relatively large contact areas with the glass substrate utilized during imaging. These large contact areas indicated that the prepared vesicles have a relatively low area expansion modulus [34], consistent with previous literature for PBd-PEO [12] and comb-type PDMS-PEO vesicles [7,24].

PDMS_12_-PEO_46_/PBd_33_-PEO_20_ mixtures also produced vesicles with unilamellar membrane structures in which the Nile Red dye appeared to be relatively uniformly distributed (Figure 2D). It is important to note, however, that the apparent homogeneity in Nile Red staining within the hybrid vesicle membranes cannot be definitively interpreted as membrane homogeneity. Indeed, it is likely that at least some degree of phase separation exists within the hybrid membranes given that PDMS and vinyl-containing hydrocarbons (such as PBd) tend to display limited miscibility [35].

### 3.2. Vesicle Area Expansion Modulus

Micropipette aspiration, a technique that has been used extensively in both lipid and copolymer vesicle measurements [8,15,30,36,37,38,39,40,41,42,43,44,45,46,47], was undertaken to quantitatively evaluate the mechanical properties of unilamellar PDMS_12_-PEO_46_/PBd_33_-PEO_20_ hybrid vesicles. Multilamellar vesicles—which would be expected to display integer multiples of the area expansion modulus values observed for corresponding unilamellar vesicles—were easily distinguished by microscopy imaging (Supplementary Figure S1) and excluded from analysis. Resulting data are representative of at least eight unilamellar vesicles of each PDMS_12_-PEO_46_/PBd_33_-PEO_20_ formulation.

**Figure 3:**
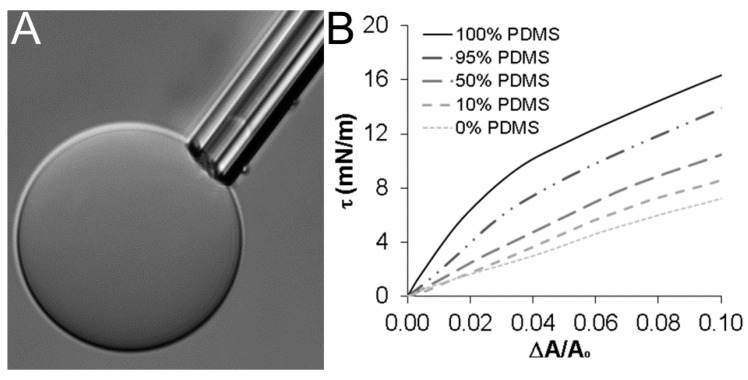
(**A**) A representative image of a 100% PDMS vesicle undergoing micropipette aspiration; (**B**) Representative tension-strain curves for pure PDMS_12_PEO_46_ and PBd_33_PEO_20_ vesicles and their mixtures through 10% areal strain. Curves for the 75% PDMS and 25% PDMS formulations are not shown for the purpose of clarity given their substantial overlap with the 50% PDMS curve.

Figure 3A shows a PDMS_12_-PEO_46_ vesicle undergoing micropipette aspiration. Representative tension-strain curves are displayed for a subset of vesicle formulations in Figure 3B. The area expansion modulus (K_A_) of each vesicle type was determined by evaluating the initial slope of corresponding tension-strain curves in the “high tension” regime. Specifically, the tension-strain curves can be described by the following equation: $\frac{\Delta A}{A}=\frac{k_{B}T}{8\pi k_{c}}\ln\left(1+c\tau A\right)+\frac{\tau }{K_{A}}$, where k_B_ is the Boltzmann’s constant, k_c_ is the bending modulus, and c is a constant of magnitude ≈0.1 [30]. In the “low tension” regime, this equation can be approximated as ln(τ) ≈ $\left(\frac{8\pi k_{c}}{k_{B}T}\right)\left(\frac{\Delta A}{A}\right)$. In the “high tension” regime, the dynamics of the tension-strain relationship shift, and the equation linking tension and strain can be approximated as τ ≈ K_Α_$\left(\frac{\Delta A}{A}\right)$, enabling K_A_ to be estimated [30].

For each vesicle type examined, the transition to the “high tension” regime occurred at $\frac{\Delta A}{A}$ ≈ 0.01 (Supplementary Figure S2), consistent with previous micropipette aspiration studies for PBd_46_-PEO_26_ vesicles [12]. In the “high tension” regime ($\frac{\Delta A}{A}$ > 0.01), tension-strain plots of pure PDMS_12_-PEO_46_ vesicles revealed an initial linear response, followed by a subsequent non-linear response (Figure 3B). Varying degrees of non-linearity were similarly observed in the “high tension” regime of each PDMS-PEO/PBd-PEO hybrid vesicle (Figure 3B), although the degree of non-linearity generally decreased as PBd-PEO levels increased. In contrast, the tension-strain response of pure PBd_33_-PEO_20_ vesicles was approximately linear in the “high tension” regime, in agreement with previous literature for PBd_46_-PEO_26_ vesicles [12].

**Figure 4:**
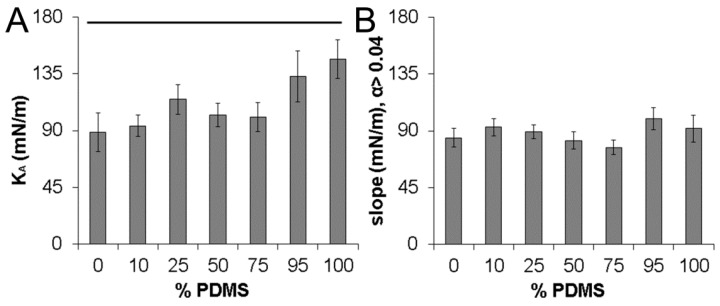
Comparison of tension-strain behavior in the “high tension” regime across vesicle formulations. (**A**) Vesicle area expansion modulus (K_A_)—defined as the slope for the tension-strain curve over 0.01< $\frac{\Delta A}{A}$ < 0.04. The black bar above the data indicates that the K_A_ of the 100% PDMS vesicles is significantly different from that of the 0% PDMS vesicles; (**B**) The average slope of the various tension-strain curves for $\frac{\Delta A}{A}$ > 0.04. No statistically significant differences were noted in the slopes of the various formulations for $\frac{\Delta A}{A}$ > 0.04. At least 8 vesicles were evaluated for each vesicle formulation in assessing each property.

In prior vesicle literature in which non-linearity has been observed [13], the area expansion modulus has been defined as the slope of the initial linear segment of each tension-strain curve in the “high tension” regime. As a result, the slope of each tension-strain plot from 0.01 < $\frac{\Delta A}{A}$ < 0.04 was taken as the K_A_ in the present studies. PDMS_12_-PEO_46_ vesicles displayed an average K_A_ value of 145 ± 16 mN/m, approximately 1.6-fold greater than the average K_A_ value of 89 ± 20 mN/m measured for pure PBd_33_-PEO_20_ vesicles (*p* = 0.020; Figure 4A). The average K_A_ values for the various PDMS-PEO/PBd-PEO hybrid vesicles were intermediate between these extremes, with the average K_A_ showing a general decrease as PBd-PEO levels increased (Figure 4A). Beyond $\frac{\Delta A}{A}$ ≈ 0.04, the tension-strain curves for PDMS-PEO containing vesicles transitioned at to a lower average slope (Figure 3B). Indeed, the average slope for each vesicle formulation over $\frac{\Delta A}{A}$ > 0.04 measured between 85–100 mN/m (Figure 4B).

The K_A_ values obtained herein for the pure PBd_33_-PEO_20_ vesicles (K_A_ ≈ 89 ± 20 mN/m) are similar to the K_A_ of 90–107 mN/m previously measured for PBd_46_-PEO_26-30_ vesicles [9,12,14]. However, the higher K_A_ associated with pure PDMS_12_-PEO_46_ vesicles relative to the PBd_33_-PEO_20_ vesicles was unexpected given the high flexibility generally associated with PDMS chains [11,18,19] and given the K_A_ of ≈92–95 mN/m previously determined for comb-type PDMS-PEO vesicles of similar molecular weight [7,24]. We do not currently have an explanation for the higher than expected K_A_ for vesicles formed from linear PDMS_12_-PEO_46_ or for the non-linearity of the associated tension-strain curves. That said, the measured K_A_ values for the 100% PDMS and 0% PDMS vesicles are reasonably consistent with the estimated interfacial energies for PDMS-PEO and PBd-PEO copolymers with similar hydrophobic block lengths: ≈32–35 mN/m for PDMS_10-14_-PEO [18] and ≈27 mN/m PBd_46_-PEO [9] block copolymers in water. In particular, the K_A_ for a vesicle membrane is often first approximated from the interfacial energy (γ) of the amphiphile in water according to K_A_ ≈ 4γ. This approximation indicates that PDMS_12_-PEO_46_ vesicles should have K_A_ values of ≈130–140 mN/m and that PBd_33_-PEO_20_ vesicles should display K_A_ values of ≈108 mN/m, in general agreement with the present results.

### 3.3. Vesicle Critical Tension, Critical Strain, and Cohesive Energy Density

**Figure 5:**
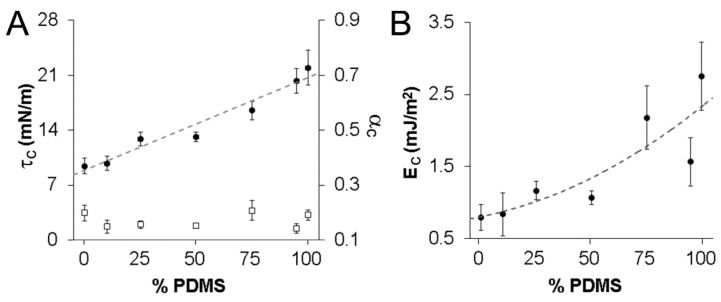
Comparison of vesicle critical tension, critical strain, and cohesive energy density. (**A**) Critical tension (τ_c_, black circles) and critical strain (α_c_, open squares); and (**B**) cohesive energy density (E_c_) across vesicle formulations. The critical tension increased in an approximately linear manner (*r*^2^ = 0.95) from 0% PDMS to 100% PDMS vesicles (*p* < 0.001). In contrast, no statistically significant differences in critical strain were noted across formulations. The cohesive energy density curve in (B) is fit by a quadratic function (*r*^2^ = 0.74). Data are shown as mean ± standard error of the mean. At least 8 vesicles were evaluated for each vesicle formulation in assessing each property.

Further examination of the tension-strain curves allowed estimation of the average critical tension (τ_c_) and critical strain (α_c_) which each vesicle type could withstand prior to rupture (Figure 5A). Vesicle τ_c_ increased in an approximately linear manner from 9.4 ± 1.0 mN/m to 22.0 ± 2.2 mN/m as the wt% of PDMS_12_-PEO_46_ in the copolymer mixture increased from 0% to 100% (*p* < 0.001). Notably, the τ_c_ of ≈22.0 mN/m measured for the linear PDMS_12_-PEO_46_ vesicles was substantially greater than the τ_c_ of ≈7.5 mN/m measured for vesicles formed from comb-type PDMS-PEO of similar molecular weight [24]. This difference in τ_c_ may be due to the difference in structure between linear and comb-type PDMS-PEO. Importantly, however, the τ_c_ for linear PDMS_12_-PEO_46_ vesicles agreed well with the ≈20 mN/m ultimate tension previously reported for a separate, “tough” copolymer vesicle system—PEO-poly(ethylethylene) (PEO_40_-PEE_37_) [8]. Similarly, previous studies of PBd_46_-PEO_26-30_ vesicles [14,48] and of PBd_125_-PEO_80_ vesicles [13] have found τ_c_ values of ≈16–20 mN/m and ≈33 mN/m, respectively. Given the observed decrease in τ_c_ with decreasing PBd length, a τ_c_ value of ≈13 mN/m would be estimated for PBd_33_-PBD_20_ vesicles, in reasonable agreement with the τ_c_ of ≈9.5 mN/m observed herein.

In contrast to τ_c_, no statistically significant differences in α_c_ were observed across the examined vesicle formulations (*p* = 0.33, Figure 5A). In brief, pure PDMS_12_-PEO_46_ vesicles displayed an α_c_ of 0.19 ± 0.02, and pure PBd_33_-PEO_20_ vesicles demonstrated an average α_c_ of 0.20 ± 0.03. Although α_c_ values for the hybrid PDMS-PEO/PBd-PEO vesicles appeared to vary from ≈0.15 to ≈0.21, these apparent differences fell below statistical significance (*p* = 0.33; Figure 5A). Thus, further study would be required to determine if true differences exist among vesicle formulations in terms of their critical strains. In comparing current results to existing literature, the present α_c_ value obtained for pure PBd_33_-PEO_20_ vesicles was similar to the 0.21 ± 0.02 previously measured for PBd_46_-PEO_26-30_ copolymer vesicles [9,14]. Notably, the α_c_ of ≈0.19 measured herein for linear PDMS_12_-PEO_46_ vesicles is markedly higher than the α_c_ of ≈0.075 previously found for vesicles formed from comb-type PDMS-PEO of similar molecular weight [24]. As with the difference in τ_c_, this difference in α_c_ may be due to the difference in structure between linear and comb-type PDMS-PEO. Importantly, the α_c_ for all the vesicles examined herein significantly exceeded the α_c_ ≤ 0.05 generally associated with lipid vesicles [49].

In addition to α_c_ and τ_c_, cohesive energy density (E_c_) is another measure of membrane toughness. For vesicles with fluid membranes, E_c_ can be estimated as the integral of the tension with respect to areal strain to the point of failure. For the vesicles in the present study, E_c_ increased over 3-fold (from ≈0.79 mJ/m^2^ to ≈2.75 mJ/m^2^) as PDMS_12_-PEO_46_ levels increased from 0% to 100% (*p* = 0.003, Figure 5B). This increase appeared to be non-linear in nature, increasingly modestly with increasing PDMS_12_-PEO_46_ for the 0%, 25%, and 50% PDMS formulations, but increasing more rapidly for PDMS_12_-PEO_46_ levels beyond 50%. In comparing the present results to previous literature, all measured E_c_ values exceeded the upper E_c_ value of 0.5 mJ/m^2^ generally associated with phospholipid membranes [50]. In addition, the 100% and 75% PDMS vesicles met or exceeded the E_c_ value of 2.2 mJ/m^2^ associated with PEO_40_-PEE_37_ membranes [8], which are considered to be tough and durable. These results are also consistent with a previous study evaluating the resistance of PBd-PEO vesicles and comb-type PDMS-PEO vesicles to burst failure due to osmotic stress [11]. In short, PBd-PEO vesicles generally failed following initial membrane rupture, whereas PDMS-PEO vesicles were observed to burst, then reseal and swell again. Carlsen *et al.* hypothesized that the ability of PDMS-PEO vesicles to repeatedly reseal following membrane disruption may be due to the greater flexibility generally associated with PDMS-PEO chains [11]. Thus, the greater E_c_ values associated with higher PDMS-content vesicles may result from the ability of flexible PDMS chains to partially “cover” or “seal” small defects in membrane structure introduced by applied tensile stress. This capacity to “seal” defects would be expected to be diminished with increasing PBd-PEO content. 

Hybrid vesicles based on mixtures of two or more copolymers have not been extensively examined in previous literature. However, hybrid vesicles formed from copolymer-lipid mixtures have been the focus of several recent studies [3,7,25,48]. Notably, Chen *et al.* [7] investigated the membrane mechanics of vesicles formed from mixtures of comb-type PDMS-PEO copolymer and the lipid DPPC (1,2-dipalmitoyl-*sn*-*glycero*-3-phosphocholine). These hybrid vesicles displayed similar K_A_ values as pure comb-type PDMS-PEO vesicles, but showed a reduced tendency to rupture relative to pure DPPC vesicles. In addition, hybrid vesicles have been formed from mixtures of PBd-PEO and the phospholipid, hydrogenated soy phosphatidylcholine (HSPC) [25]. Resulting hybrid vesicles displayed a substantially increased area expansion modulus relative to pure PBd-PEO vesicles, although membrane tension and strain at rupture were not assessed. Similarly, Nam *et al.* examined vesicles formed from graded mixtures of the lipid POPC (1-palmitoyl-2-oleoyl-*sn*-*glycero*-3-phosphatidylcholine) and PBd_46_-PEO_30_ copolymer and found that membrane K_A_, critical tension, and critical strain were each modulated by increasing POPC levels [48]. The present data are consistent with these previous lipid-copolymer hybrid vesicle results in that the examined hybrid copolymer vesicles displayed mechanical properties intermediate between that of each pure copolymer vesicle type.

Limitations of this current work include that the degree of inhomogeneity in the membrane composition and organization of the hybrid vesicles was not assessed. This is significant as inhomogeneity in membrane composition, both between and within specific hybrid vesicle membranes, has the potential to substantially impact vesicle properties [32,51]. However, good reproducibility was generally observed between the aspiration tension-strain plots for separate vesicles formed from the same PDMS-PEO/PBd-PEO mixture. Specifically, the degree of variation in the tension-strain responses of each hybrid vesicle was similar to that observed for the pure vesicle controls, as evidenced by comparison of the standard errors associated with their respective K_A_, τ_c_, and α_c_ values. These observations indicate that the individual vesicles within a specific PDMS-PEO/PBd-PEO vesicle population have similar overall compositions. Furthermore, these observations suggest that the length-scale of potential inhomogeneity within the vesicle membranes formed from PDMS-PEO/PBd-PEO mixtures may be less than the tip diameter of the selected microaspiration pipettes (≈10 µm). An additional limitation of the present work is that the membrane thickness of each vesicle type was not confirmed—rather, previous literature was relied on to estimate membrane thickness. Furthermore, future work must be conducted to assess the structural basis of the non-linear mechanical response, the high K_A_, and other remarkable aspects of linear PDMS_12_-PEO_46_ vesicle behavior.

## 4. Conclusions

In the present work, we fabricated hybrid vesicles from mixtures of linear PDMS-PEO and linear PBd-PEO with the long-term goal of enabling broad, application-specific tuning of vesicle membrane fluidity and permeability for nano/micro- sensor, reactor, and drug carrier applications. However, given that importance of membrane expansion modulus, strength, and toughness in each of these applications, the mechanical properties of the resulting hybrid vesicles had to first be confirmed. Toward this end, we characterized the mechanical behavior of vesicles formed from graded mixtures of linear PDMS_12_-PEO_46_ and linear PBd_33_-PEO_20_ in the high tension regime. PDMS_12_-PEO_46_ vesicles displayed higher K_A_ values than PBd_33_-PEO_20_ vesicles, while also showing an increased capacity to absorb stress and energy prior to failure. However, the strain to failure was similar for both of these vesicle formulations. PDMS_12_-PEO_46_/PBd_33_-PEO_20_ hybrid vesicles generally displayed graded responses in between that of pure PDMS_12_-PEO_46_ and pure PBd_33_-PEO_20_ vesicles. Thus, the hybrid vesicles retained or exceeded the strength and toughness characteristic of PBd-PEO vesicles, indicating that future assessment of PDMS-PEO/PBd-PEO vesicle membrane permeability and fluidity may be warranted.

## Supplementary Files

Supplementary File 1
