# Supplementary material for: Mechanical Characterization of Hybrid Vesicles Based on Linear Poly(Dimethylsiloxane-b-Ethylene Oxide) and Poly(Butadiene-b-Ethylene Oxide) Block Copolymers
Source: Sensors (Basel). 2016 Mar 18;16(3):390. doi: 10.3390/s16030390 (PMC4813965; doi:10.3390/s16030390)
Supplement: Supplementary File 1 [file sensors-16-00390-s001.pdf]

Supplementary Material: Mechanical Characterization of Hybrid Vesicles Based on Linear Poly(Dimethylsiloxane-b-Ethylene Oxide) and Poly(Butadiene-b-Ethylene Oxide) Block Copolymers

Jeffery Gaspard, Liam M. Casey, Matt Rozin, Dany J. Munoz-Pinto, James A. Silas and Mariah S. Hahn

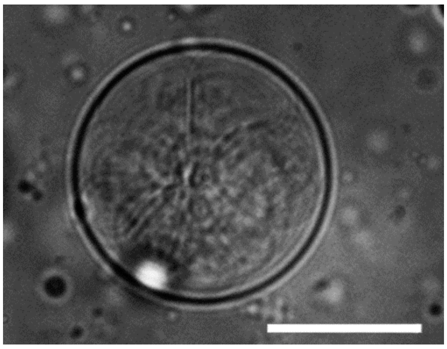

Figure S1. A zoomed-in image of a multilamellar PBd-PEO vesicle observed within the vesicle population shown in Figure 2A; scale bar = 50 μm.

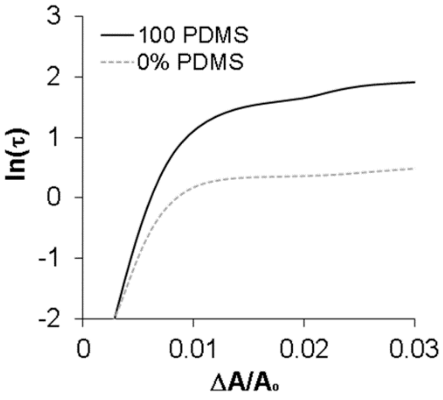

Figure S2. Representative ln(τ) versus strain curves for pure $PDMS_{12}PEO_{46}$ (100% PDMS) and pure $PBd_{33}PEO_{20}$ (0% PDMS) vesicles at low strains. Note the transition in the slope of the ln(τ)-strain curves occurring at ≈1% areal strain, indicative of a transition from the "low tension" regime to the "high tension" regime. Assessment of the bending modulus associated with this low tension regime was not conducted as greater resolution within this region would have been necessary to yield accurate bending modulus values.
